# R-spondin 3 governs secretory differentiation in the gastric oxyntic glands

**DOI:** 10.1172/JCI163380

**Published:** 2022-11-01

**Authors:** Ken Kurokawa, Timothy C. Wang, Yoku Hayakawa

**Affiliations:** 1 Department of Gastroenterology, Graduate School of Medicine, University of Tokyo, Tokyo, Japan.; 2 Division of Digestive and Liver Diseases, Department of Medicine, Columbia University, New York, New York, USA.

## Abstract

The gastric oxyntic glands are maintained by gastric stem cells that continuously supply all differentiated cell types within the corpus epithelium. Stem cells are supported by stromal cells that make up the stem cell niche. In this issue of the *JCI*, Fischer et al. report on their use of genetically engineered mouse models and organoids to study the role of R-spondin 3 (RSPO3) in the stomach. RSPO3, one of the major stem cell niche factors, primarily promoted secretory differentiation in the normal stomach, but also contributed to regeneration following injury. Mechanistically, RSPO3 was upregulated in the stroma by loss of chief cells and then activated the YAP pathway in gastric stem and progenitor cells, which appeared to be critical for regeneration of the secretory lineage. These data substantially advance our understanding of the regulation of gastric stem cells and highlight a function for RSPO3 in the gastrointestinal tract, which is as the gatekeeper of secretory differentiation.

## Stem cells in the gastric oxyntic glands

The stomach can be subdivided into three different regions: the antrum, the cardia, and the corpus, the latter being the acid-secreting portion that makes up the majority of the stomach. The corpus epithelium consists of the oxyntic glands that contain a variety of secretory cell types, including parietal cells secreting gastric acid, chief cells secreting digestive enzymes, and mucous neck cells secreting gastric mucins and antibacterial peptides. Similarly to gastrointestinal (GI) glands at other sites, the oxyntic glands are maintained by long-lived stem cells that have the ability of self-renewal and multipotentiality. While most GI stem cells reside near the base of glands, corpus stem cells are confined to the upper part of glands, namely the isthmus, and supply progeny bidirectionally toward both the top and base of the glands. The corpus gland base is occupied by abundant chief cells, most of which are thought to be derived from mucous neck cells located in the midglandular region. Thus, one major route of cell supply in the oxyntic glands is the downward migration starting from isthmal stem cells, in which daughter progenitors first differentiate to mucous neck cells and then later transdifferentiate to the basal chief cells (1).

## Stromal R-spondins in the gastric stem cell niche

GI stem cells are supported by surrounding stromal cells that secrete numerous growth factors, regulating the growth and differentiation of the glands. R-spondins have long been considered one of the critical factors needed to sustain GI stem cells. R-spondins are secreted proteins that bind to the Lgr receptor family and are thought primarily to stabilize Wnt receptors and enhance Wnt signaling. In previous studies, Sigal et al. showed that one R-spondin, R-spondin 3 (RSPO3), was highly expressed in *Myh11*^+^ myofibroblasts near the gastric gland base and modulated proliferative responses to injury (2, 3). Indeed, Lgr5 is expressed by cells at the gland base throughout the GI tract, in close proximity to the R-spondin signal from the adjacent stromal niche. While Lgr5-expressing cells have been implicated as stem cells elsewhere in the GI tract, in the corpus, Lgr5 is expressed predominantly by chief cells near the gland base and not much by cells in the isthmus (4). Lgr5 expression by chief cells raises questions as to the primary role of RSPO3 signaling, given its long association with stemness, self-renewal, and Wnt signaling. Indeed, a previous study by the group suggested that in the gastric antrum, RSPO3 acted primarily to induce differentiation of basal Lgr5^+^ cells into secretory cells (3).

## RSPO3 regulates differentiation and regeneration in the corpus

**Figure 1:**
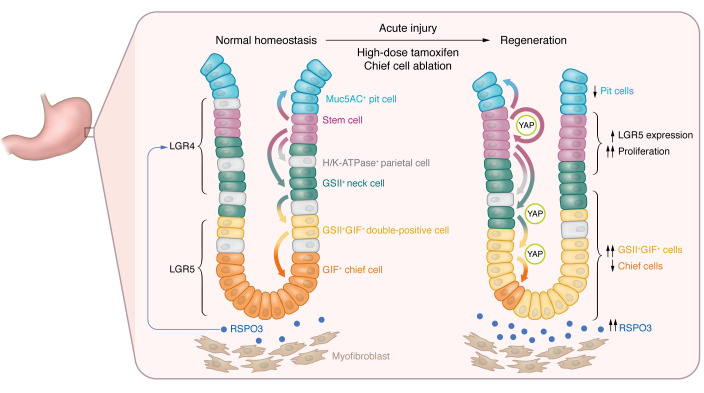
RSPO3 modulates secretory differentiation in the oxyntic glands. Stem cells residing at the corpus isthmus supply daughter cells bidirectionally. Surface pit cells are supplied upwards from the isthmus, while other cell types, including mucous neck, chief, and parietal cells, are supplied downwards. Under normal homeostasis, stromal myofibroblasts secrete RSPO3 and regulate cellular differentiation from isthmus stem cells, presumably via the Lgr4 receptor. Thus, RSPO3 overexpression leads to an increase in chief and parietal cells and a decrease in surface pit cells. Loss of chief cells, induced by acute mucosal injury or genetic ablation, triggers upregulation of RSPO3 in myofibroblasts. In the regenerative state, upregulated RSPO3 activates stem cells, induces their proliferation and expansion, and promotes secretory differentiation to restore the depleted cell lineages (i.e., chief and parietal cells).

In this issue of the *JCI*, Fischer et al. (5) report on the role of RSPO3 in the corpus glands under normal homeostasis and following injury, which they undertook by modulating its expression in transgenic mouse lines. They found that overexpression of RSPO3 increased glandular height and the numbers of parietal cells and chief cells, but decreased the number of surface pit cells, while KO of *Rspo3* led to the opposite effects. In *Rspo3*-KO mice, GSII^+^ mucous neck cells were instead increased, consistent with impaired differentiation toward the chief cell lineage. Surprisingly, KO of *Rspo3* did not impair epithelial proliferation around the isthmus in normal homeostasis. Therefore, under normal conditions, the primary function of RSPO3 appears to be the regulation of corpus differentiation, modulating pit versus secretory cell fate, rather than stimulating proliferation (Figure 1) (5).

In contrast, following acute stomach injury resulting from high-dose tamoxifen (HDT) or genetic ablation of chief cells using diphtheria toxin (DT) in *Lgr5*-DTR mice, depletion of RSPO3 was found to inhibit mucosal proliferation and regeneration, leading to delayed recovery of the chief cell population (5). It has previously been shown that both chief cells and parietal cells are decreased by HDT-induced gastric injury and that HDT- or DT-mediated chief cell ablation results in a marked expansion of proliferating neck progenitors during regeneration (4, 6). Importantly, the authors showed that loss of chief cells following acute injury appears to be the main trigger leading to RSPO3 upregulation. In addition, they demonstrated that regeneration of chief cells occurred independently of Lgr5^+^ chief cells, presumably from corpus progenitors, as shown previously (4, 6). RSPO3 upregulation following injury resulted in marked corpus proliferation primarily in the isthmus and subsequent recovery of the chief cell compartment; however, in mice deficient in RSPO3, fewer chief cells were generated and the gland base comprised primarily GSII^+^ mucous neck cells, suggesting that RSPO3 is critical for chief cell regeneration derived from neck progenitors (Figure 1).

## Potential role of RSPO3 in gastric preneoplasia

The research group also studied a more chronic *Helicobacter pylori* (Hp) infection model and found that chronic Hp infection together with RSPO3 overexpression led to robust mucosal hyperplasia and proliferation. Mechanistically, the authors concluded that RSPO3-induced mucosal regeneration and hyperproliferation depends on the activation of the yes-associated protein (YAP) pathway, which previously has been shown to be involved in intestinal stem cell activation and mucosal regeneration (7) as well as gastric tumorigenesis (8, 9). Thus, RSPO3-dependent YAP activation plays a central role in gastric regeneration during the acute and chronic injury phases. However, in the chronic Hp infection model, KO of *Rspo3* did not further diminish chief cell numbers and RSPO3 overexpression did not restore chief cells, suggesting that the loss of chief cells observed in Hp-associated gastric atrophy may be due to alterations outside of RSPO signaling.

In addition, Fischer and colleagues suggest a relationship between RSPO3 and spasmolytic polypeptide–expressing metaplasia (SPEM), although these data need cautious interpretation. The markers used for chronic SPEM (i.e., GSII^+^GIF^+^ double positivity) are more problematic in the setting of acute injury where this expanded lineage is short-lived. Such double-positive cells, as shown again in this study, exist in the normal gastric glands as intermediate cells between neck and chief cells during the normal process of chief cell differentiation. While RSPO3 did lead to an increase in GSII^+^GIF^+^ cells, consistent with the process of expanding chief cells, the notion that RSPO3 contributes to SPEM development was not supported by studies in *Rspo3*-KO mice, which showed the same level of GIF^+^GSII^+^ cells as WT mice, and thus seemed inconsistent with a major role in SPEM. The data perhaps point to some other bottleneck to full chief cell differentiation. In addition, given that there are no true cancers in the models the authors used, the role of RSPO3 in gastric cancer development remains uncertain at this point (5).

## Conclusions and future directions

Multiple studies in the past revealed that disruption of RSPO (Lgr) signal impairs mucosal homeostasis, particularly following injury and in regenerative states (10–12), and R-spondins appear essential for long-term GI organoid cultures (13, 14), suggesting that R-spondin/Wnt signaling may be critical for sustaining GI stem cells. Fischer et al. (5) nicely highlight a different function of R-spondin — promotion of corpus epithelial differentiation toward the major secretory lineages (chief and parietal cells) and away from the pit lineage. This role is quite consistent with their earlier antral study (2) as well as with work reporting that RSPO3 promotes differentiation of intestinal Paneth cells (15, 16).

While KO of *Rspo3* did not appear to impair corpus stem cell function, overexpression of RSPO3 did induce hyperproliferation in the isthmus where Lgr5 was not highly expressed. While the authors did not investigate the mechanisms here very deeply, several clues were provided (5). In the antrum, RSPO3 stimulated proliferation by Axin2^+^Lgr5^–^ stem cells via Lgr4 (17), and thus one might postulate that Lgr4 is involved in the proliferative response to RSPO3 in corpus progenitors. However, the authors show that Lgr5 may also be involved in isthmal proliferation. Fischer et al. (5) observed low levels of Lgr5 in the normal isthmus region, but found a marked increase in Lgr5 following injury, consistent with previous reports (4). Thus, isthmal stem cells may very well express both Lgr4 and Lgr5, such that in injury models of the corpus, Lgr5 should not be used as a specific chief cell marker.

The models presented (5) should allow further investigation of questions not fully addressed in this study. Is Lgr4 required for the proliferative response and for the increase in Lgr5 expression in the corpus isthmus? Does deletion of Lgr4 inhibit mucosal proliferation or secretory differentiation, and is either Lgr4 or Lgr5 involved in corpus neoplastic development? What is the mechanism by which acute chief cell loss leads to RSPO3 upregulation in the stroma? How are RSPO3 and their receptors expressed in the stomach chronically infected with Hp? Finally, the role of R-spondin signaling in secretory differentiation versus stem cell proliferation should be studied in other parts of the GI tract. In conclusion, Fischer et al. (5) have unraveled unique functions of RSPO3 in secretory differentiation and regeneration, and their findings should guide future studies into the pathogenesis of human gastric diseases.

## Version 1 11/01/2022

Electronic publication
